# Analysis and Interpretation of metagenomics data: an approach

**DOI:** 10.1186/s12575-022-00179-7

**Published:** 2022-11-19

**Authors:** Gauri S. Navgire, Neha Goel, Gifty Sawhney, Mohit Sharma, Prashant Kaushik, Yugal Kishore Mohanta, Tapan Kumar Mohanta, Ahmed Al-Harrasi

**Affiliations:** 1 Department of Microbiology, Savitribai Phule Pune University, Pune, Maharastra 411007 India; 2 Department of Genetics and Tree Improvement, Forest Research Institute, 248006 Dehradun, India; 3 Inflammation Pharmacology Division, Academy of Scientific and Innovative Research (AcSIR), CSIR-Indian Institute of Integrative Medicine, Jammu-180001, Jammu Kashmir, India; 4 Department of Molecular Medicine, Medical University of Warsaw and Malopolska Center of Biotechnology, Karkow, Poland; 5 Independent Researcher, Valencia, Spain; 6 University of Science and Technology Meghalaya, Baridua, 793101 Meghalaya India; 7 Natural and Medical Sciences Research Center, University of Nizwa, Nizwa, 616 Oman

**Keywords:** Metagenomics, Next-generation sequencing, Crops, Pipelines, Analysis

## Abstract

Advances in next-generation sequencing technologies have accelerated the momentum of metagenomic studies, which is increasing yearly. The metagenomics field is one of the versatile applications in microbiology, where any interaction in the environment involving microorganisms can be the topic of study. Due to this versatility, the number of applications of this omics technology reached its horizons. Agriculture is a crucial sector involving crop plants and microorganisms interacting together. Hence, studying these interactions through the lenses of metagenomics would completely disclose a new meaning to crop health and development. The rhizosphere is an essential reservoir of the microbial community for agricultural soil. Hence, we focus on the R&D of metagenomic studies on the rhizosphere of crops such as rice, wheat, legumes, chickpea, and sorghum. These recent developments are impossible without the continuous advancement seen in the next-generation sequencing platforms; thus, a brief introduction and analysis of the available sequencing platforms are presented here to have a clear picture of the workflow. Concluding the topic is the discussion about different pipelines applied to analyze data produced by sequencing techniques and have a significant role in interpreting the outcome of a particular experiment. A plethora of different software and tools are incorporated in the automated pipelines or individually available to perform manual metagenomic analysis. Here we describe 8–10 advanced, efficient pipelines used for analysis that explain their respective workflows to simplify the whole analysis process.

## Background

Microorganisms are omnipresent and hence have an immense effect on the biosphere of the Earth. All organisms, from humans to plants, impact the microorganisms in their vicinity [1]. For instance, they are the catalyst in maintaining a healthy relationship between man and the food (i.e., the crops) he eats to flourish and evolve. This thought raises many questions about microbial populations hidden in the soil, which indirectly contribute to human health but are unknown due to our inability to culture them. There was no approach to this thought until, in 1998, Jo Handelsman coined the term 'Metagenomics' – which has the potential to reveal the secrets of the microbial world. She described it as the cloning and functional analysis of collective genomes of soil microflora to be the metagenome of the soil. In her article, she also stated that: not all soil microflora is culturable; hence, soil alone has a huge number of untapped microbial communities yet to be explored [2]. Since then, it has developed as a platform with the broadest applications in molecular biology [3]. It is as if Handelsman has given us the key to unlock the mysteries of the microbial world. This key is currently being applied to study the microbiome of various environments, from the human gut to the sea floor [4]. The science of metagenomics can extensively help us to understand the relationship of the crop with the microbes present in the soil. The soil is a rich source of microorganisms, but when it comes to crops, the rhizosphere is like the coral reef of the soil where all the significant microbial species reside. We discuss further in the review metagenomic studies on rhizosphere and its respective crops [5] (Table 1).

**Table 1:** Analysis of recent literatures on crop plants and their studies associated with rhizosphere

Analysis of crop literature					
**Sr. No**	**Title/Ref**	**Crop**	**Aim/Objective**	**Software/tools used**	**Interpretation/outcome**
1	[106]	Wheat	To characterize the rhizospheric microbiome of high Zinc (HZn) and Low Zinc (LZn) wheat; (2) To determine microbes that can mobilize zinc. And (3) Identify the abundance of microbes	1.Bowtie 2 v2.2.7 2.MEGAHIT v1.1.3 3.Prodigal v2.6.3 4.MMseqs2 Linclust kaiju v1.7.3	Novel microbial species with zinc mobilizing potential were identified as Massilia and Pseudomonas species, which may have a functional module to accelerate soil zinc mobilization. Around 30 novel bacteria were isolated using high throughput targeted culturomics
			Involved in zinc mobilization in the given environment and their role in altering the concentration of grain zinc among the cultivars	eggNOG-Mapper v2.0.1	These isolates having strong potential to increase the availability of Zn in soil could be used as a synthetic community to improve the nutrition and growth of cereal plants
2	[107]	Legumes	To analyze the rhizospheric microbiota assembly of two lentil cultivars under the effect of rice fallow ecology to identify the diversity of the microbial population	1.WINRHIZO software package 2.MeV tool 3.Parallel-metatool	The two cultivars Farmer-2 and Moitree show cased insignificant differences in the diversity and role of microbiomes concerning Nitrogen metabolism in their rhizosphere
3	[108]	Legumes	To determine the effect of strigolactones on the recruitment of microbes to the rhizosphere	1.LefSe software 2. Mothur software	Altering the strigolactone signaling and biosynthesis pathway alters the rhizosphere bacteria community
4	[109]	Wheat and chickpea	To understand the microbiome, present in the detritus sphere and the effects it shows due to agricultural management that involves soil tillage and crop rotations	1. SoapAligner software 2.MetaGeneMark software 3. CD-HIT software 4.BLAST software	When the rhizosphere and detrituspheric microbiomes collaborate in the presence of decaying roots, it is observed that the rhizospheric microbiome degrades the plant root exudates, and the specific genes corresponding to membrane transporters, amino acid, and carbohydrate metabolism enhance their expression
5	[110]	Wheat	To study the response of chemical fertilizers on putative PGPR richness present in the commercial Cadenza, the wheat variety is grown in a low input agricultural soil exhausted in most nutrients. Under such conditions, the beneficial microbes are known to have a key role in sustaining the crop growth and production	CASAVA data analysis software (Illumina)	The rhizobacteria present in the soil is beneficial to plants as it is involved in insoluble nutrients mobilization in soil but in the presence of chemical fertilizers, its population is decreased considerably
6	[111]	Wheat	To study the collective response of eCO2 and nitrate levels on the function and structure of the bacterial community attached to the root surface	1.StepOne software v2.3 (Applied Biosystems) 2.CD-HIT-EST v4.8.1 3.MEGAN v6.15.2 4.Trinity mapping v2.8.4 5.Cytoscape v.3.7.2	The combined effect of CO2 and nitrate levels are responsible for plant growth and development and is also benefit the growth and function of the root surfaceassociated bacterial population which is involved in the fitness of root and root colonization
7	[112]	Chickpea	To analyze endophytic bacterial communities for their functionality and diversity present in internal root tissues of native legumes species growing into different locations of south Portugal further assess its potential to accelerate plant development and growth	Tree of Life (iTOL) v4	The association of rhizobia and specific non –rhizobial endophytic bacteria elevates the growth of chickpea majorly via increasing the nodulation and nitrogen fixation capacity shown by mesorhizobial strains
8	[113]	Sorghum	To determine the time dependent change in the microbial complexity in the rhizospheres of field-grown sorghum	USEARCH software suite	An OUT of bacterial origin from the genus Pseudomonas was identified in the microbiome of the rhizosphere of Sorghum bicolor. The species *Pseudomonas* was never before reported to be associated with the said plant
9	[114]	Rice	To investigate the factors involved in chemotaxis systems that have been affected by the selection at the time of domestication of rice species	–-	Genes involved in bacterial chemotaxis showed a greater richness in the rhizospheres of wild rice as compared to cultivated rice, and the two types of rice showed significant variation in the compositional makeup of their respective chemotaxis genes
10	[115]	Rice	Root tissues of the rice (Oryza sativa L.) is habited by Bacillus paralicheniformis strain KMS 80 (MTCC No. 12704) that is known to display plant growth promoting abilities along with nitrogen fixation. Whole genome sequencing is performed on the DNA of this bacterium to evaluate its functional gene profile	–	Whole genome sequencing of the Bacillus species results were predicted as: 21 genes took part in Nitrogen Metabolism pathway and two main genes glnR and tnrA (transcriptional factors) were involved in regulating nitrogen fixation in the *Bacillus* strain KMS 80. This will help to understand the enhanced endophytic nitrogen fixation and other beneficial role of *B. paralicheniformi*s in rice
11	[116]	Rice	To determine the potential effect of Nitrogen fertilizer and the Azospirillium product on growth and development of rice and its yield and on diversity of its bacterial community with respect to both the rice roots and the rhizosphere	Silva database, QIIME (version 1.7.0)	Greater Nitrogenase activity (533–634 nmol C2H4/plant/h compared to the control) was observed when the Azospirillum product (A was applied with less than 1 dose of the nitrogen fertilizer. The grain yield was (6,001.3– 6,480.6 kg/ha) which is same as adding 100% of nitrogen fertilizer. The richness of bacterial community of the soil rhizosphere was higher as compared to the rice roots as predicted by metagenomic analysis
12	[117]	Wheat		1.Trimmomatic v 0.36 2.Pear v 0.9.6 3.VSEARCH v 2.7.1 4. QIIME 2 v 2019.4	The analysis showed that application of P fertilization for a long term affected the soil Carbon and Phosphorus significantly and phoD-harboring bacterial community compositions in rhizosphere of wheat soil. This high application of P fertilizers decreased the total Bacterial OTUs along with diversity and their connections potentially affecting the biogeochemical cycles of the soil
13	[118]	Legume	The soil bacterial community was assessed due to addition of P fertilizer on the soil to determine the difference in the diversity and structure of the clover rhizosphere microbial community along with the nitrogen fixing bacterial communities of the soils in the presence of different Hg contamination levels	Quantitative Insights into Microbial Ecology	High levels of Hg in the soil decreased bacterial diversity and community abundance along with increased in the richness and diversity of nitrogen-fixing bacteria. High Hg level soils had the presence of Rhizobium which was a biomarker. Other important factors affecting structure and abundance of the microbial community was: soil total nitrogen (TN), soil organic matter (SOM), nitrate nitrogen (NO3 − N) and available molybdenum (Mo)
14	[119]	Cowpea, sorghum, maize	To determine the mineral nutrient concentrations, activity of P- enzyme and the differences in the rhizospheres of intra-hole planted cowpea (cvs. TVu 546 and PAN 311), maize (cv. ZM 521), and sorghum (cv. M48) with their sole cropped	QIIME, UCLUST	Modifications in the rhizosphere bacterial community structure were observed because of intra-hole cropping. The soil was dominated by Proteobacteria, Actinobacteria, Verrucomicrobia, Acidobacteria, Firmicutes, Bacteroidetes and Planctomycetes which together consisted more than 95% of the sequence. Absent for the soil were pathogens like like Ralsotonia and Agrobacterium

## Applications

As mentioned earlier, metagenomics is a versatile branch of science, having two basic approaches: (A) taxonomic application (sequence-based analysis) and (B) Functional application (Function driven analysis) or a combination of both, depending on the requirement of the objective.

### Taxonomic application

This approach is used to find out the phylogenetic relationships of the sequenced gene with taxonomic groups of microorganisms known in the database. In this case, the phylogenetic clusters like 16S rRNA gene sequence are targeted where operational taxonomic units (OTUs) [6] are compared against their amplitude to estimate the microbial species abundance in that particular environment [7].

One such application of metagenomic analysis involves taxonomic profiling and identification of plant pathogens [8] using next-generation sequencing along with disease diagnostics, microbiome analyses, and outbreak tracing [9]. Taxonomic profiling is also used in metabarcoding (similar to metagenomic analysis), which has the potential to identify all the microbes, including rare and abundant taxa [8, 10].

### Functional application

This approach is used to find a sequence with a functional gene having a particular activity or if the gene is novel having a specific function in a functional pathway [11, 12]. This is achieved by shotgun metagenomics, which includes whole-genome sequencing, which further involves functional annotation of a gene [13, 14]. Functional annotation is divided into two steps: gene prediction and gene annotation, where gene prediction helps to find the potential protein sequences. After identification, these sequences encoding the protein are compared with protein families in databases and annotated functionally by matching the family's function [15].

Functional metagenomics broadly identifies novel proteins/genes that contribute to the microbial population's function and affect the environment [16]. For example, soil collected from different parts would identify novel antimicrobials like Terbomycine A and B, novel anti-infectives like lactonases, and bacterial NHLase [17].

## Road map to metagenomics study

Following are the fundamental steps involved in a metagenomics experiment where each step has significance.

### Step 1: sample collection

This is the very first and essential step to begin a metagenomics project where the particular sample to be examined is selected and used for DNA isolation. The sample collection time points may vary depending on the study and are expected to be used freshly to isolate DNA [14, 18, 19]. For instance, the microbial community in the peanut plant rhizosphere was analyzed by collecting the sample around the roots during the nodulation period [20]. In another example, infants' fecal samples from the Oukasie clinic were collected to analyze the enteric RNA virome in the northwest province of South Africa [21]. The participants were immunized with the Rotarix vaccine, after which samples were collected at three different time points [21]. Another study analyzed freshwater lakes for their metagenomics content [22]. This conveyed the importance of time-period, geographical conditions, and pre-treatments present during sample collection.

### Step 2: isolation of DNA

The method for isolating DNA from the sample collected has to be chosen appropriately because it would result in erroneous results, and highly reliable knowledge would be lost. The selection of the method depends on the type of sample collected [14, 19, 23]. Environmental and human-sourced samples have many microbial cells belonging to different phyla/classes [19]. Because of this, the sample contains heterogeneous cells as far as the genomic contents, architecture, and morphology of their cell wall are concerned. It becomes mandatory to process the samples first and then add lysis reagents to extract a sufficient quantity of good-quality community DNA. In the experiment, three enzymes, lysozyme, lysostaphin, and mutanolysin, can brake either 1,4-beta glycoside linkages or transpeptidase bonds present in Gram-positive and Gram-negative bacteria cell walls and assist in spheroplast formation. The Spheroplast formed is extremely liable and breaks easily in the presence of lysis reagents, physical pressure, or mechanical forces [24].

### Step 3: NGS library preparation and sequencing

One of the critical steps in the NGS workflow is preparing the DNA for sequencing, i.e., creating an NGS DNA library which is a collection of similarly sized DNA fragments with known adapter sequences added to the 5' and 3' end of the sequence.

The isolated DNA is subjected to library preparation which consists of 4 basic steps as follows:

1. DNA fragmentation/Target selection
2. Adapter sequences
3. Size selection
4. Final library quantification and QC

The isolated DNA is fragmented using physical or enzymatic methods (whole genome), or if the sequence of the specific target sequence in the fragment is known, PCR amplification of these known fragments is done to produce DNA amplicons (16S rRNA target sequence is extensively used) within the desired size range. Next, the specific DNA adapter sequences are annealed (ligated) to these fragments at the 3' and 5' ends. These double-stranded adapters are around 20-40 bp fragments with known sequences. One adapter contains the primer annealing site, and the other adapter is used for anchoring the DNA fragment to a surface for sequencing. For example, beads or a solid surface containing a complementary DNA sequence. The size selection of the ligated DNA fragments is made by gel electrophoresis (PACBIO SMRT bell Express Template Preparation Kit), columns (Qiagen), or magnetic beads [25]. If the size of the targeted DNA fragment is known, then the magnetic beads become a better option as compared to gel electrophoresis. Quantifying the library is the last crucial step which is checked on a Bioanalyser system, giving information on the concentration of the library and the different fragment size lengths present. Quantitative real-time PCR (qPCR) is another method for quantification and is known to give the precise quantity of the library but is unable to estimate the library size [26]. Based on the requirement of the experiment, the DNA library is prepared by either amplicon fragmentation or whole genome fragmentation and forwarded for sequencing.

#### Targeted sequencing (amplicon sequencing)

The targeted sequencing approach is the most extensively applied strategy to characterize microbial populations. The basic technique used in this method is DNA isolation. PCR amplification is performed with polymerase chain reaction primer sets that individually target a taxonomically informative gene common to eukaryotes and prokaryotes.

#### 16S rRNA gene amplicon sequencing

16S rRNA sequence is considered the most conserved taxonomic marker (bacterial) as it is sequenced in considerably less time. Hence, it is a gold standard for extensive phylogenetic analysis [27]. The same technique is used for metagenomic studies to identify the sample's taxonomic profile of microbial communities. There are approximately 1500 base pairs (bp) in size, with nine highly conserved regions and nine variable regions (V1–V9) in the complete 16S rRNA. The conserved regions of the genes are used for primer binding during PCR amplification, whereas the hypervariable regions are used for identifying sequence diversity in prokaryotes [28]. Platforms like Illumina sequencing use V3 and V4 regions to obtain the taxonomic classification by comparing these regions with those already known and available on large public databases like NCBI [29], SILVA [30], GreenGene [31], RDP [32], and 16S rRNA gene. Gene sequencing is the most widely used approach to disclose the identity of the pathogen as they are signature-specific sequences in bacterial species with higher accuracy. Bacterial wilt disease in *Cucurbita maxima* in China caused by *Ralstonia solanacearum* was identified using 16S rRNA gene sequencing of the isolates collected from the plants infected by wilt disease [33]. A recent study with 16S rRNA gene amplicon metagenomic analysis resulted in the identification of a rhizospheric microbial community of plants like *Eichhornia crassipes* [34] and mangrove species (*Sonneratia alba, Rhizophora mucronata, Ceriops tagal*, and *Avicennia marina*) [35].

#### ITS sequencing

Internal transcribed spacer (ITS) of the nuclear ribosomal DNA is used to identify the eukaryotes in the particularly fungal community in the metagenomic samples. The isolated DNA sample is subjected to PCR with primers specific to regions of 5.8S and LSU rRNA, flanked by the ITS2 region [36]. The Library of the amplified DNA is then used for sequencing, for instance, the Illumina MiSeq platform. The generated sequenced data is analyzed using PIPITS. The first pipeline with complete bioinformatics automation is wholly devoted to sequencing ITS regions belonging to fungal origin. The PIPITS_PROCESS part of the pipeline uses the VSEARCH tool for clustering sequences into OTUs [36]. These OTUs are further processed to build OTU tables, which are the final interpretable results of the analysis. Recent studies used high-throughput Internal Transcribed Spacer Amplicon Sequencing to analyze field-grown maize and soybean microbiomes from southeastern and central Brazil [37]. They identified degrader bacteria and fungi of rhizosphere soil from a toluene phytoremediation site [38].

#### Whole genome sequencing (shotgun metagenomics)

Shotgun sequencing is another method used in characterizing the abundance of microorganisms available in a particular environment. This method not only identifies the microbial species but also can generate information about the genes (including 16S rRNA) present in the metagenomic sample. This approach offers information about the functional characterization of the genes belonging to the microbial communities in the sample. This method is PCR independent, where there is no chance of biasing due to primer binding. This factor is helpful for finding unknown microbes in the sample, which may otherwise not be detected by targeted sequencing [39]. This method also helps identify and discover novel viruses in the given environment. Indeed, broad-range genetic markers are unavailable for viruses; shotgun sequencing has developed the technique to identify viruses. Recent studies with shotgun sequencing include assessing the functional genes of maize rhizosphere microbiota, which were found to be diverse. Genes involved in nitrogen fixation, phosphate solubilization, quorum sensing, trehalose and siderophore production, phenazine biosynthesis, daunorubicin resistance, acetoin, and 1aminocyclopropane-1-carboxylate deaminase were reported [40]. Both the sequencing mentioned above are performed by anyone method of next-generation sequencing technologies: nanopore technology, sequencing by synthesis, pyrosequencing, sequencing by ligation, and single-molecule real-time sequencing, ion torrent sequencing [41].

### Step 4: metagenomic sequence data analysis

After the sequencing is done, this is the critical part of the entire experiment where the sequenced data generated will include multiple samples with billions of sequence data reads. Hence, the data needs to be trimmed down to a meaningful nucleotide sequence supporting the stated hypothesis to pull out sensible and reasonable information. To analyze big data, different software is developed and devoted to a particular function.

#### A. Taxonomic analysis

The metagenomes are analyzed by comparing them with sequences already present in the databases or by a particular activity. For example, the software DOTUR is developed to study the operational taxonomic units, thus predicting the richness of the microbial population present in the given environment [42]. There are automated pipelines developed for the complete analyses of the metagenome where a series of software are applied together step by step to achieve interpretable results. For example, Metagenomic Rapid Annotation using subsystem Technology (MGRAST), a platform available on the web, is programmed for processing, analyzing, and sharing metagenomic data [43]. Details about the pipelines and software/tools will be discussed further in the review. A Recently developed pipeline CAMAMED a composition-aware mapping-based metagenomic pipeline, is used for both taxonomic and functional profiling levels. The pipeline was used to check the taxonomic profile of gut microbiota from colorectal adenoma and colorectal carcinoma individuals. The result predicted a significantly changed gut species ratio to 2.67% of the total 374 species [44]. ezTree, a computational pipeline, is developed to automatically identify single-copy marker genes for a group of genomes and build phylogenetic trees from the marker genes. ezTree was tested on a group of proteobacteria species which revealed that ezTree was highly influential in pinpointing marker genes and constructing reliable trees for different groups of bacterial genomes [45].

#### B. Functional analysis

When the metagenomic data are studied for identifying genes and enzymes of a particular function, it is a function-driven analysis. Such studies are paramount as they may reveal any possible novel enzymes or pathways. For such analysis, pipelines are designed like the DMAP (Dragon Metagenomic Analysis Platform). The platform annotates and comparisons of genomic or metagenomic sequence data via its Annotation and Compare Modules [46]. There is a repertoire of different pipelines for such annotations and comparisons available currently, which are highly efficient with such an enormous quantity of sequenced data. Recently developed FMAP, a functional mapping and analysis pipeline, which is not used for functional profiling but is also used for pathway analysis (for example, Crohn's disease) which listed ten pathways significant to the phenotype of Crohn’s disease [47]. A couple of pipelines used in functional profiling are MOCAT2 for metagenomic assembly and annotation [48] and MetaStorm for customizable metagenomic annotation of target genes [49].

## Milestones in metagenomics

To understand the journey of metagenomics through the years, we have summarized the important milestones that constitute the metagenomics era. The diagram represents the time of metagenomics from when Leeuwenhoek reported oral microbiota in 1676 to the milestones achieved in 2019 in the human genome project. We describe here the objectives of the latest significant milestones achieved in the past five years (since 2015) around the world.

### Ocean sampling day (2015)

The ocean sampling day was an initiation taken and organized under the funding provided by European Micro B3 (Marine Microbial Biodiversity and Bioinformatics) to get a picture of the marine microbial biodiversity along with the role played by oceans of the world concerning the microbial communities. It was considered the worldwide mega-scale sequencing drive to generate the most extensive standardized microbial data set acquired in one day. The study aims to analyze marine microbial community composition and functional traits. Researchers all over the globe were successful in obtaining a generous amount of environmental metadata that included precisely 150 metagenomes along with 18S/16S rRNA amplicon sequence data sets [50].

### Host-targeted drugs affect microbiota populations (2015)

The study states that the use of commonly consumed medications affects the gastrointestinal microbial richness and their respective gene expression, which would affect human health positively or negatively concerning drug treatment. The proton pump inhibitors (xenobiotics) were studied and checked for their effect on the microbiota of the lumen of the GIT. Xenobiotics are reported to change the functions and gene expression of the dynamic microbiome of the human gut [51].

### Human skin microbiome (2016)

The work studied the coherent analysis of bacterial, fungal, and viral species that interpreted the site-specificity of the microbiota and individual signatures [52].

### Human microbiota affects cancer therapy (2018)

The gut microbiota is involved in altering the body's response to a cancer patient.

(melanoma, advanced kidney, or Lung cancer) against the drug treatment. For instance, the Gut microbiome regulates the efficiency of PD-1-dependent immunotherapy targeting epithelial tumors [53].

### Genomes assembled using metagenomics anticipate unusual characterization of microbiota associated with humans (2019)

Metagenomic analysis showed the presence of an unknown uncultured bacterial candidate present in the human body where the individuals belonged to different ages, geography, and lifestyle [54]. It can be said that the gut has a novel set of microbiomes that expands the phylogenetic divergence of the human metagenomic database [55]. The above examples give a glimpse of the diversity of research done. Each year, numerous research articles are added to the prior art on metagenomic studies on various topics. Every year, the study parameters of the metagenomic analysis are evolving, unfolding the various branches of applications metagenomics can serve us. One such application is the interplay of the microbial community in the rhizosphere and its effect on the health and development of the crop.

## Metagenomics and crops

The plant Microbiome is an active community of microorganisms associated with a particular plant. A plant's microbiome is divided into two parts: (A) Microbes inhabiting the atmospheric section of the plant are known as the Phyllosphere, and (B) Microbial communities inhabiting the below-ground portion of plants are called the rhizosphere. It is the fraction of soil beneath the root secretions we, as science students, have studied since our school days. It is estimated that rhizosphere soil can nurture approximately 10^11^ microbial populations for every gram of soil collected [56], along with precisely 30,000 prokaryotic species [57]. Due to the discharge and intake of a diverse array of chemicals/compounds from the soil, various groups of microorganisms can be metabolically active [58], thus making the rhizosphere the most active niche of the soil [59].

The diversity of Rhizosphere soil can be classified into six classes, namely (I) bacteria, (II) viruses, (III) archaea, (IV) fungi, (V) algae, and (VI) protozoa and their abundance in the rhizosphere are in the decreasing order with Bacteria is the most abundant of them all (10^8 -^ 10^9^ g^-1^). This group of unicellular organisms, together with the plant roots, forms the most complex habitat on Earth [60]. Thus, we focus on the hotspot of the soil, i.e., the rhizosphere. The involvement of rhizospheric microbial species with the plants marks a significant area for conducting metagenomic research. Understanding the metagenomic analysis of such interactions can be valuable in various agriculture applications like crop rotation and soil tillage, levels of nutritionally essential elements present in the soil, etc. The below table states different objectives studied with metagenomics in recent years. It gives us an overview of the depth and direction of the research currently being pursued.

The diagram below illustrates the overview of the study performed on the Pea plant rhizosphere [61]. This study aimed to check how the Pea plant affects the soil's microbial community and can shape its rhizosphere microbiome. The study was done with two types of soils (I) pea plant rhizospheric soil and (II) bulk soils with nutrients in the form of fertilizer. Metagenomic analysis was done by amplifying the V4 region of the bacterial 16S rRNA gene using universal bacterial primers. This particular research article was chosen to get insights into the latest studies on the legume rhizosphere. In the figure, the dotted arrows represent the elevation/enhancement offered by one element towards another, and the inhibition arrows depict the inhibition provided by an element for the other. The green arrows explain that the microbial species/phyla are observed in abundance in the presence of the pea plant rhizosphere. The red arrows explain that the presence of the particular microbial species has decreased in the presence of the pea plant rhizosphere.

The following part of the article will explain the details and technicalities of metagenomic studies, including next-generation sequencing, software, and metagenomic analysis workflows (Table 1). The microbial species *Chloroflexi* and *Nitrospirae* are seen to be decreasing in the presence of pea plant rhizosphere because they are slow growers and are unable to cope in front of other fast-growing microbes present (star marked) [61].

## Sequencing platforms

Within the last decade, the cost of sequencing the exome of a human has decreased approximately 15,000 times, going from 15 million USD to 1000 USD (https://www.genome.gov/27565109/the-cost-ofsequencing-a-human-genome/). Michael L. Metzker explains that we should not focus on DNA sequencing technology. Still, we should expand the limitations of the research we do to collect sufficient complex data which can apply to interpret the answers to numerous questions simultaneously [62]. One of the examples of such thoughts is metagenomics, where the sequencing technologies are similar, but the ability to create high throughput data and analyze it is different. It can answer several complex questions at the same time. This brings our review to study important sequencing platforms that have evolved through time and experience.

The utilization of DNA sequencing has changed over time. Earlier it was answering simple questions like a sequence of nucleotides and predicting the gene function by homology studies. Later, it became the ultimate tool for understanding taxonomic and phylogenetic relationships between different organisms. This was possible due to the decreased sequencing costs that supported sequencing and comparing thousands of genomic sequences for microbial taxonomic studies [63]. One such field that uses NGS to identify microbes is microbial diagnostics, where even epidemics can be traced on a real-time basis [63].

NGS has many applications in the field of molecular biology and biomedical sciences. Currently, it is massively used in metagenomic studies where complex analysis of microbial communities is performed to answer critical scientific problems. Complex investigations of metabolic characteristics of bacterial communities or even symbiotic development of bacteria and the host have been possible in recent years [64]. The discovery of new bacteria by metagenomics can eventually lead to the discovery of novel antibiotics. Further ahead, the availability of whole-genome sequences can be used for analyzing the metatranscriptome of the microbiota and its interactions in the gut [65] or, in our case, the rhizosphere, and the crop plants. The below table explains the technicalities of each sequencing platform available currently in the market with their respective manufacturers.

## Software’s/tools used in the metagenomics analysis

The above tasks require a large amount of processing power and storage capacity and a thorough understanding of using computational methods from many areas (information theory, signal processing, and systems science) in conjunction with one year of experience to provide trustworthy findings. Therefore, metagenomic analysis systems with automated workflows for various processing purposes, combining tools in the form of services operative inside processing pipelines, are in high demand (Table 2). Several analytic pipelines have been created for metagenomic research (Table 2). Several pipelines have been developed to analyze single-organism genomic data [66–68]. When using NGS for metagenomic analysis, however, the limits of comparable methods created for single organism data have been exposed for purposes of metagenomic research.

**Table 2:** Summary of next-generation sequencing platforms available in the market with their respective manufacturers

Sr. No	Manufacturer	Platforms	Template preparation	Chemistry behind Sequencing	Run time (sequencing)	Maximum data output (per run)	Output read (Maximum)	Run conditions with reading lengths
1	**Roche**	454 FLX Titanium	Emulsion PCR on micro beads	Pyro-sequencing	10 h	450 MB	1Million per plate	Modal 450 bp, max.600 bp read lengths
		454 FLX +			23 h	700 MB	1Million per plate	Modal 700 bp, max.1000 bp read lengths
		454 GS Junior			10 h	35 MB	0.1 Million per plate	∼450 bp read lengths
2	**Illumina**	Illumina GAIIx	Flow cell surface used for Bridge-PCR	Reversible terminator sequencing-bysynthesis	6 days	900-1000 GB	250 Million per lane	HiSeq2000/2500 (high output mode): max.2 × 125 bp read lengths
		Illumina HiSeq1000			40 h	250–300 GB	125–150 Million per lane	HiSeq 2500(rapid run mode): max.2 × 250 bp read lengths
		Illumina HiSeq1500			55 h	13.2–15 GB	22–25 Million per flow cell	MiSeq: 2 × 300 bp read lengths
		Illumina HiSeq2000			29 h	100–120 GB	400 Million per flow cell	NextSeq: 500(high output mode) with max. 2 × 150 bp read lengths
		Illumina HiSeq 2500			26 h	32–39 GB	130 Million per flow cell	NextSeq 500 (midoutput mode) with max. 2 × 150 bp read lengths
		Illumina MiSeq			8 h	15 GB	25 Million per flow cell	2 × 300 bp read lengths
		Illumina NextSeq550			12–30 h	120 GB	400 Million	2 × 150 bp read lengths
		Illumina HiSeqX			< 3 days	1.6–1.8 Tb	2.6–3 Billion	2 × 150 bp read lengths
		Nova Seq 6000			13-44 h	80-6000 Gb	1.6–40 Billion	2 × 250 bp read lengths
	**Life Technologies**	SOLiD4	Emulsion PCR was done on micro-beads; PCR on Flow Chip surface for the 5500 W models	Sequencing-by-ligation	8 days	260 Gb	160 Million per lane	
		SOLiD5500			8 days	320 Gb	256 Million per lane	
		SOLiD5500xl						SOLiD 5500 xl: max.60 bp + 60 bp read lengths
		SOLiD5500W						
		SOLiD5500xl W						SOLiD 5500xlW:max.2 × 5 0 bp read lengths
	**Life Technologies**	Ion PGM	Emulsion PCR on microbeads	Semiconductor dependent sequencing-by-synthesis	7.3 h	1.2–2 Gb	4–5.5 Million per chip	IonPGM(318): max.400 bp read length Ion Proton(318): 200 bp read length
	**Life Technologies**	Ion Proton	Emulsion PCR on microbeads	Semiconductor dependent sequencing-by-synthesis	2-4 h	Upto10Gb	60–80 Million per chip	IonPGM(318): max.400 bp read length Ion Proton(318): 200 bp read length
	**Pacific Biosciences**	PacBioRS	Information not provided	Single-molecule, real-time DNA sequencing-by-synthesis	2–3 h per cell	400 Mb per cell	0.05 per SMRT cell	Chemistry used C2/P4 ~ 8000 bp mean read length
	**Oxford Nanopore**	Flongle	Information not provided	Nanopore sequencing	1 min-16 h	2.8 Gb	126 channels per flow cell	> 4 Mb read length
		MinIONMk			1 min-72 h	50 Gb	512 channels per flow cell	> 4 Mb read length
		PromethION			1 min-72 h	14 Tb	2675 channels per flow cell	> 4 Mb read length
		GridIONMk			1 min-72 h	250 Gb	512 channels per flow cell	> 4 Mb read length

### Pipelines for metagenomics analysis

To analyse metagenomic sequencing data, bioinformatics programs like CloVR-metagenomics, [66] (ii) Galaxy platform (metagenomics pipeline) [69, 70] (iii) IMG/M [71, 72] (iv) MetAMOS [73], (v) MG-RAST [74, 75] (vi) RAMMCAP [76], and (vii) Smash Community [76] are available (Table 3). They are very much efficient in effectively analyzing the metagenome data.

**Table 3:** Display of features of current bioinformatics pipelines for metagenomic data analysis

Tasks/Pipeline	Quality control	Assembly	Gene detection	Functional annotation	Taxonomic analysis	Comparative analysis	Data management
Clover metagenomics	No	No	Yes	Yes	Yes	Yes	Yes
Galaxy platform^a^	Yes	No	No	No	Yes	Yes	No
IMG/M	No	No	Yes	Yes	Yes	Yes	Yes
MetAMOS	Yes	Yes	Yes	Yes	Yes	Yes	Yes
MG-RAST	No	No	Yes	Yes	Yes	Yes	Yes
RAMMCAP	No	No	Yes	Yes	Yes	Yes	No
Smash Community	Yes	Yes	Yes	Yes	Yes	Yes	No

^a^Refers to the metagenomics pipeline of galaxy

### CloVR-Metagenomics

Two distinct inputs are required to run CloVR-metagenomics (CloVR: Cloud Virtual Resource), desktop software for automating sequence analysis. The raw sequencing data (in fasta format) and the metadata file (tab-delimited) with sample-specific information for comparative analysis are required. Booting from their website needs a Virtual Machine (VM) player, which is free. Visitors to Amazon Cloud can establish a cloud-based instance and utilize the Request Instances Wizard to discover an accessible Amazon Machine Image (AMI). As a first step, the process employs UCLUST [77] to cluster duplicate sequence reads and then conducts BLAST [78] homology searches against the COG [79] and RefSeq [80] databases for functional and taxonomic identification, respectively.

To discover differentially abundant characteristics, the results of the two studies are fed into the integrated Meta stats software [81]. Integrated custom scripts in R language (The R Project for Statistical Computing 2) are used to normalize taxonomic or functional counts for grouping and visualization. Most importantly, CloVR's design allows users to choose between local resources and cloud computing capability. Quality control and gene identification (available exclusively in the single-genome and 16S rRNA software) make the platform largely reliant on third-party software, making it more vulnerable to the read length of sequencing datasets as a possible drawback.

### Galaxy platform (metagenomics pipeline)

A general open-source framework for integrating computational tools and databases into a coherent, concerted workspace, Galaxy is being advanced for medicinal research that requires a large amount of data. Another option is for users to download and install Galaxy on their computers to fully use local resources, tools, and databases for bespoke workflows. To install locally, simply run the appropriate BASH run. sh (added to the original downloaded directory) script. The new Galaxy method for metagenomic data conducts automated analyses utilizing integrated specialist tools [82] when combined with raw sequencing results (raw reads). When used with raw sequencing reads, it executes a series of computerized studies using specialized integrated tools, according to a new Galaxy process for metagenomic datasets [82].

Those studies include:

(i) Checking the readings for quality and filtration (custom tool),
(ii) Editing text and converting data formats (custom tools),
(iii) Searching the NCBI-NT database for homology,
(iv) taxonomic research (custom tools), and
(v) Results visualization with the help of custom tools.

Most significantly, this platform enables any user to develop workflow processes by integrating any custom tools of their choice (third party or proprietary) capable of handling various analytical activities, all while offering a highly intuitive user interface. For a complete local installation, however, advanced programming skills are required, making the solution unsuitable for anyone who is not an expert.

### IMG/M

Experimental metagenome data management and analysis tool IMG/M includes a database of bacteria and other archaeal species and tools for data exploration and comparison analysis. Assembled sequence data may be searched for genes, contigs, and scaffolding, as well as their related functional characterizations, using data exploration tools. The comparative data analysis suite includes methods for (i) determining the gene content and phylogenetic profile of any metagenomic sample, including I profile-based selection tools, (ii) gene neighborhood analysis tools, and (iii) multiple sequence alignment tools. Through its web server's GUI, this platform may publish and manage a user's (Meta) genome while using its cloud infrastructure. Despite this, the user is still responsible for quality control of raw readings and assembly. IMG/M is developed only for metagenome assembly, unlike other metagenome tools. All users must have an IMG account that may seek on the IMG website.

### MetAMOS

This pipeline receives raw sequence reads or completed contigs as input and assembles them into a metagenomic dataset. The modules of this pipeline make up an entire analytical workflow that includes: (i) quality control using two different tools (FASTX-Toolkit 5, Babraham Bioinformatics - FastQC 6), (ii) sequence assembly to contigs with eight different assembly methods exploiting four different assembly tools. Several tools can be incorporated into MetAMOS processes to comprehensively analyze metagenomic datasets, including raw sequencing reads, contig, and scaffold data, which may be automated. Moreover, the lack of a user-friendly interface makes accessing its extensive collection of tools complex since all actions should be done from the Linux command-line shell. At the same time, its customization requires the use of scripts. A Python script, namely INSTALL.py, automates the complete installation procedure by obtaining the newest version and running it.

### MG-RAST

Both raw sequences read datasets, and previously assembled contigs can be used as inputs in this process. The user must register for the online service to upload metagenome datasets and create tasks.

It consists of four major tasks, which are divided into modules:

(i) Data normalization,
(ii) Finding putative protein-coding genes and coding elements by screening the sequences against public databases using predefined default search criteria.
(iii) Input data, computation of functional annotations and taxonomy designations, and (iv) Result in visualization with the SEED Viewer [83].

All job-relevant resultant data is saved in flat file and SQLite (SQLite 7) formats throughout pipeline installation to achieve the best data management based on relational database technology. Comparative metagenomic analysis of the original dataset may be performed using the results obtained from the preceding stages compared to additional metagenomes or whole genomes obtained from the SEED environment [43]. This platform, like IMG/M, provides a user-friendly GUI behind an HTTP server, making data management and analysis as straightforward as feasible. Other than that, it offers a wide range of functional and comparative genomics tools and the ability to handle assembled and unassembled data. Regardless of the absence of components for basic read quality assurance and assembly operations, the pipeline offers a practical and well-established taxonomic annotation system that fully exploits the potential of public sequence databases [84].

### RAMMCAP

Rapid Analysis of Multiple Metagenomes with a Clustering and Annotation Pipeline, RAMMCAP is a metagenomic platform that focuses on programmatic optimization to reduce the computing cost of the different processing activities. However, since CAMERA has been discontinued, the RAMMCAP pipeline is only accessible as a standalone utility for installation on your computer or laptop [76]. To install, you'll need to download the most recent package, which contains all of the necessary applications and databases [76]. After then, each pipeline's required applications must be built and installed independently before the automated pipeline can use them. CD-HIT method is used to cluster sequences from one or more metagenomic samples. This is followed by a second clustering of the protein sequences, which is done in parallel with the ORF discovery task on the raw reads, using a local algorithm (ORF finder) [76] (Li 2009).

### Smash community

Smash Community may be viewed as the metagenomic version of its predecessor SmashCell; a program developed to study single-cell amplified microbial genomes in high-throughput [85]. Users must download the current version of SmashCommunity and compile/install it using the standard BASH instructions to install the package on their system (configure, make, make install). Installation of necessary applications and databases is required before installing the pipeline. To do this, run the BASH scripts offered in the release (such as install dependencies.ubuntu.sh). Raw read files from 454 or Sanger sequencing methods are required for this process (i.e., long-read sequence data) [86]. In addition to the command-line-only package, the user must manually install the various necessary programs that make up the entire analytical pipeline. Assembler limitations are also carried down the pipeline, limiting its performance to only long-read sequencing data. This is why only long-read sequencing data can benefit from this technique (an issue that will soon be obsolete as even Illumina machines are increasing their read length output with each new sequencer release) [85]. Despite that, experts will find it an excellent choice for doing extensive and completely automated metagenomic investigations on a dedicated local server.

### YAMP pipeline

In "Yet Another Metagenomics Pipeline", AMP, an already-containerized workflow, handles shotgun metagenomics sequencing data up to the taxonomic and functional annotation stage utilizing state-of-the-art tools and software. YAMP is implemented in NextFlow [87] and is accompanied by a Docker [88] and a Singularity [89] container. The YAMP script, parameters, and documentation are available at https://github.com/alessia/YAMP.

### YAMP workflow

**Fig. 1:**
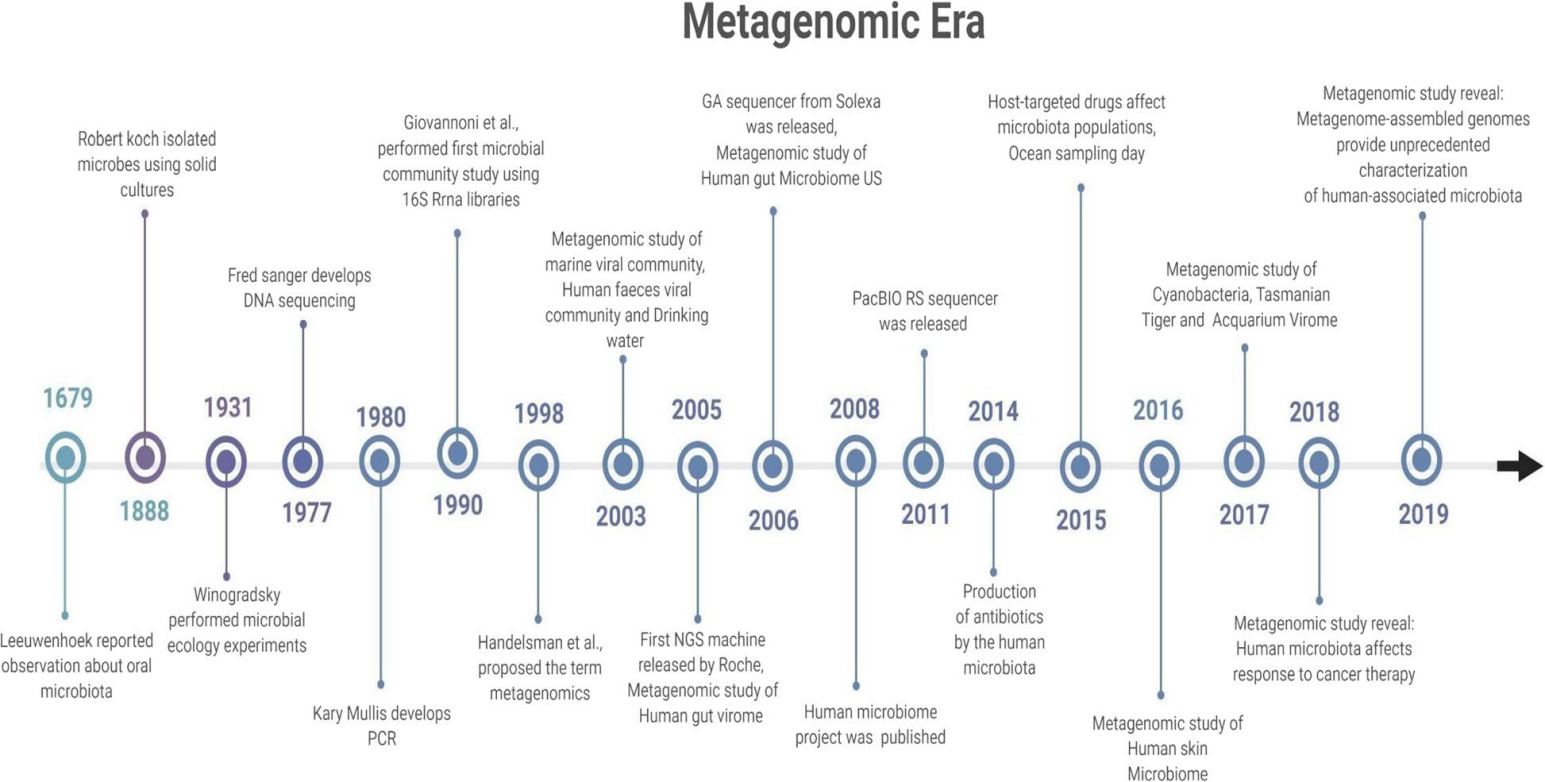
Metagenomic era highlighting the milestones in the development of Metagenomic studies achieved around the globe

Each block in the YAMP process is broken down into three parts [90]. YAMP removes all duplicates before cutting to eliminate bias introduced by reading changes. There are numerous phases of assessment and display of data quality, as shown in Fig. 1. De-duplication, an optional step in the QC process, is used to eliminate identical readings that PCR may have produced. Using PCR-free library preparation techniques (e.g., TruSeq) enables biological duplicates to be kept. It is next necessary to remove adapters, artifacts, and phiX from the reads. The reads are subsequently quality trimmed. Trimmed reads are deleted if they've gotten too short. Because they may map to several genomes or genomic areas, their presence may compromise subsequent studies [91]. If you're working with paired-end reads, you should use singleton reads (i.e., paired-end reads that have been stripped of their mates). Lastly, readings were filtered for the presence of pollutants, such as reads that do not belong to the investigated environment, before they were included in the study. Many low-complexity sequences and some characteristics (such as ribosomes) are highly conserved throughout species. They should be deleted from the custom database of contaminant reads to avoid false-positive matches. Numerous phases are included in the QC process to determine the quality of the readings and evaluate the trimming and purification steps [91]. Many procedures are then taken to estimate -diversity and characterize the microbial community's taxonomic and functional profiles, including identifying and quantifying the microorganisms present (taxonomic binning and profiling) and their functional capabilities (functional characterization) [90].

**Fig. 2:**
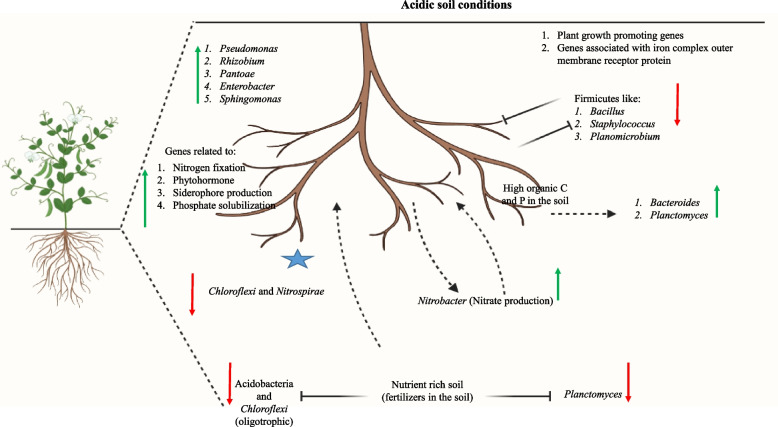
The legume rhizosphere: The Pea plant shapes its rhizosphere microbiome

**Fig. 3:**
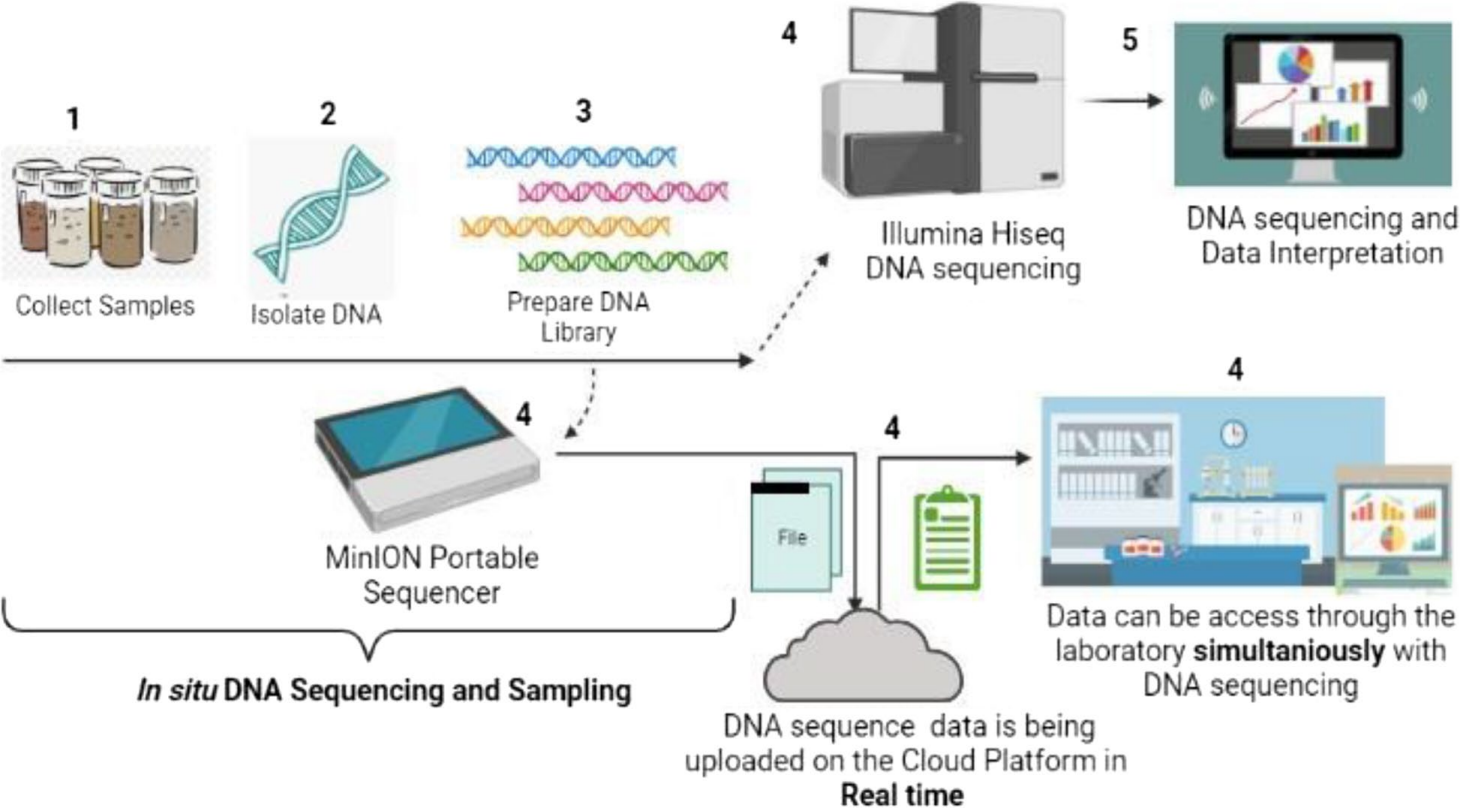
Highly Portable Sequencing Platform: Oxford Nanopore. Having understood the importance of NGS technologies in the functioning and development of metagenomics, we hereon move forward with analyzing the metagenomic data generated by the above technologies. This analysis is done using different software and tools, which are either manually used or automated by sequentially programmed to achieve different functions involved in the analysis

**Fig. 4:**
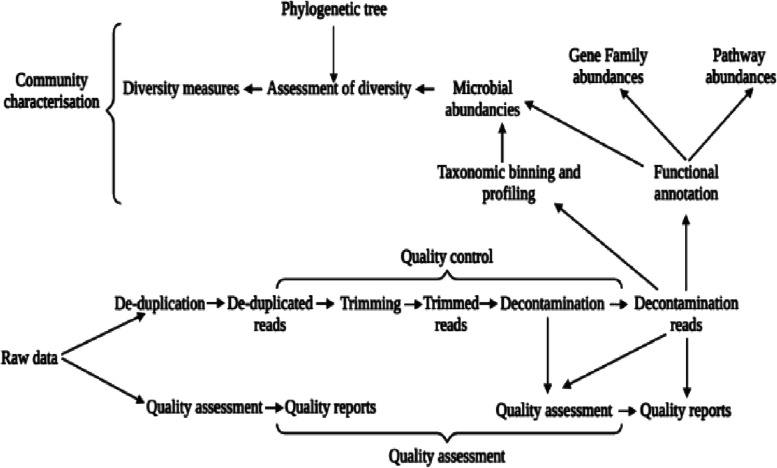
Showing the phylogenetic tree produced after YAMP analysis

### Implementation

A workflow management system, NextFlow, was utilized to create YAMP, which has been used in numerous life-science initiatives [92, 93]. Because of NextFlow's user-transparent high-level parallelization, big applications are assured of scalability. A UNIX-based system's executor allows workflows seamlessly port to any UNIX-based system (e.g., a local machine or HPC facility). In addition to YAMP, a Docker container and a Singularity container [89, 90] are installed. Platform independent virtualized operating system Docker provides all the applications required by YAMP and tracks their versions. As a result of singularity, these characteristics may be transferred to HPC systems, with which Docker is incompatible. YAMP supports both a single container and a multi-container scenario. To analyze the metagenomic data, YAMP incorporates state-of-the-art technologies [90] (Figs. 2, 3 and 4).

Several well-established programs in the BBmap suite [94] are used to perform quality control (QC) on single-end. Paired-end reads from all major sequencing platforms, including clumpify, Bduk, BBwrap, and BBduk (i.e., Illumina, Roche 454 pyrosequencing, Sanger, Ion Torrent, Pacific Biosciences, and Oxford Nanopore). Additionally, they are very scalable to big metagenomics projects and samples due to their computational efficiency. FastQC, which offers comprehensive information on reads’ quality, is employed to do QC evaluation and visualization [91].

### YAMP input/output

YAMP supports both single-end and paired-end FASTQ files as inputs for processing. Outputs provided by the program include the taxonomic composition, a relatively significant quantity of genes and pathways for microorganisms, and pathways coverage for multiple -diversities. Users may tailor workflow execution by utilizing command-line arguments or editing a simple plain-text configuration file. Users can retain temporary files, such as those created by the QC stages or during the HUMAnN2 execution if they so want [95].

### NextFlow and metaflow|mics pipelines for microbiome marker data analysis

MetaFlow|mic is a new framework for microbiome data analysis that summarizes its functioning from DADA2, Mothur, VSEARCH, and other tools into an easy-to-use set of pipelines. Beyond a simple set of commands, our pipeline is a complete system based on standards that allow for cross-platform portability, flexibility, and repeatability [96]. In addition, it contains three high-level tasks, a proprietary demultiplexing pipeline, and two end-to-end analysis pipelines, one for investigating bacterial data (16S marker) and the other for analyzing fungal data (ITS marker). To optimize the deployment of processes across different platforms, NextFlow was developed. Because of this, all the necessary software is included in each analytic pipeline to QA/QC the reads and estimate diversity at the operational taxonomic unit and the exact sequence variation levels. In addition to Nextflow, R, Python, Docker, and Singularly containers are used to spread the analytic pipelines [97]. The pipelines used in MetaFlow|mics were developed in conjunction with the Center for MICROBIOME analysis via Island Knowledge and Investigations (C-MAIKI), the Hawaii EPSCoR Ike Wai project, and the Hawaii Data Science Institute [97].

### MetaFlow|mics are composed of three distinct microbiological analysis pipelines

**Fig. 5:**
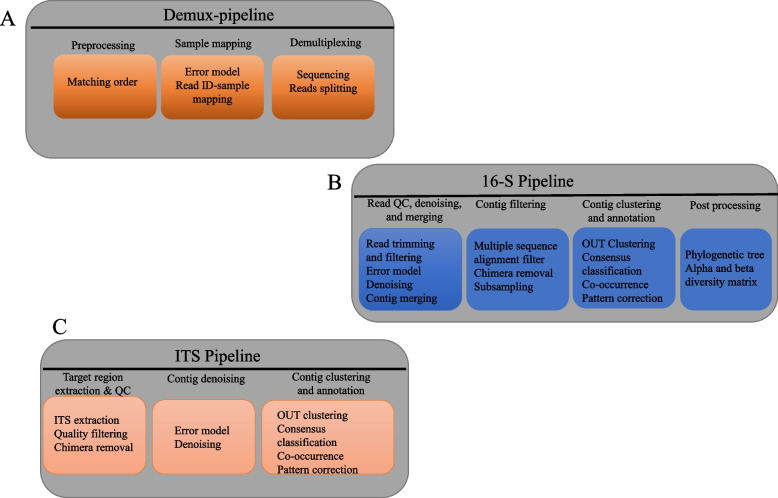
In MetaFlow|mics, the three analytic pipeline phases are shown in this diagram. **A** Demultiplexing pipeline, (**B**) 16-S pipeline, (**C**) ITS pipeline

As a result, we developed a probabilistic process for demultiplexing sequencing reads and 16S barcode pipelines for bacteria and fungus data analysis. Figure 5 summarizes the pipelines, which are further explained in detail [81]**.** It is now possible to generate terabytes of data with modern sequencing devices, far beyond the minimal throughput required for sequencing a single biological sample. Several biopsies are regularly mixed and sequenced in the same run. The samples were first identified by a DNA barcode comprising a few DNA nucleotides (A, C, G, and T) [98]. To demultiplex, or unpool, the DNA sequences created by the sequencing apparatus, the index found at the beginning of each DNA sequence must be read and placed into the proper sample file. Using a unique probabilistic approach, the MetaFlow|mics demultiplexing barcode parts that do not match any of the known barcodes can be recovered using the script. Using NextFlow's domain-specific language, the demultiplexing parallelization of pipelines is possible regardless of infrastructure.

### Pipeline implementation

A large amount of computing power is required to analyze modern metagenomic studies containing millions of reads. In microbiome experiments, the supply of such materials varies from project to study, which is unfortunate. As part of our pipeline, we're working to make it easier to deploy and parallelize it on high-performance settings, such as HPC clusters or cloud computing services [97].

### Reproducibility

Through containerized computing, a major focus of MetaFlow|mics is durability. In the case of an analytic pipeline and a dataset, the description of the program and the active Operating System (OS) are the major causes of variance in the findings. Each pipeline's computing environment may be set up using Dockerfiles, which includes all of the necessary deployment information, such as operating system type and version and application versions. As a result, Dockerfiles are used to faithfully replicate any environment as a standalone container for each analysis. They provide consumers with a transcript that can be shared and reused. Prior pipeline versions may be accessible on GitHub for backward compatibility concerns, giving the user a means of switching back to previous run parameters if necessary [97].

### Scalability

Parallelization is effective and resource-efficient since many pipeline modules handle each sample individually. By seamlessly moving data across computers and gathering outcomes from several actions performed simultaneously (or linearly if resources are limited), NextFlow simplifies parallelization and makes it easier to use [87]. Deployment on high-performance computing clusters (SLURM and SGE, for example) and cloud settings, the pipeline includes pre-configured configuration files (Google Cloud). Instance: in a supercomputer system, the user can specify queue names for each task under the pipeline, or machine types can be assigned automatically in specific scenarios based on established default values. Cloud computing's fine-grained resource allocation can speed up the runtime and save expenses because of its fine-grained resource allocation. MetaFlow|mics can automatically scale up when unexpected resource consumption arises in a process [96]. An unfinished or overly memory-intensive procedure will be resubmitted [97].

### Flexibility

Because implementation environments are independent of the source code used for analytical logic, as was explained earlier in this article. They may thus be used safely in a multi-user system where user privileges are limited. Users need just install Docker (or Singularity) on their computer. Most shared systems, such as HPC clusters and cloud services, come pre-installed with these frameworks, which is not uncommon [97].

### Monitoring

Especially when identifying abnormalities in the execution of the pipeline or monitoring the run progress, pipelines might provide outputs that are difficult to read and comprehend. As a result of this, MetaFlow|mics offer two different forms of execution results [96]. It is also possible to produce a series of data visualizations (such as heat maps, scatter plots, and box plots), which graphically represent the results [97].

### SqueezeMeta, a highly portable, fully automatic metagenomic analysis pipeline

When it comes to studying huge numbers of metagenomes or metatranscriptomes, SqueezeMeta is a very flexible pipeline [99]. Everything from assembly through taxonomic/functional assignment of the resultant genes to abundance estimate is provided. Because it uses a sequential metagenomic assembly and later contig merging, SqueezeMeta can run on moderately-sized computing infrastructures, alleviating the stress of co-assembling tens of metagenomes. For processing MinION sequences, the program comprises specialized software and modifications [99]. Some of SqueezeMeta's sophisticated features set it apart from previous pipelines, such as the following.

1. Use of co-assembling and read mapping for estimating gene abundances in each metagenome.
2. It’s possible to process an endless number of metagenomes by combining separate metagenomes using a different co-assembling method.
3. The ability to do nanopore long readings.
4. To get individual genome, binning and bin checking must be used.
5. An internal check on the contigs and bins taxonomy annotations.
6. Metatranscriptomic support is provided by mapping cDNA readings to reference metagenomes or co-assembling the two.
7. Results may be stored in a MySQL database and then exported, shared, or viewed from anywhere using a web interface [100]

SqueezeMeta is designed to analyze several metagenomes in one go. This program has three different operating modes to choose from the figure.

Sequential mode: There is a sequential analysis of all metagenomes. Binning is not used in this mode, as each metagenome is processed individually [99].

Co-assembly mode: After that, the data from all samples are blended and assembled into one single data set. Once the co-assembling is completed, reads from individual samples are mapped back to the co-assembling. Contigs can be classified into genomic bins based on their abundance [101, 102].

Merged mode: because it is a computationally-intensive process, co-assembly takes an enormous amount of random access memory (RAM). If the number of samples is large, the computer infrastructure may not be able to meet the demands. While in the merged mode, SqueezeMeta allows for the assembly of a large number of samples, utilizing a technique similar to that of TARA Oceans and binning to extract the maximum number of genomes from the samples [99].

## Conclusion and future prospective

Next-generation Sequencing and Metagenomic analysis/Interpretation are the two most dynamic technologies that constitute Metagenomics. These two technologies are the backbone and the soul of this field of study. Metagenomics applications in crop sciences are humungous and can solve the mysteries that can improve crop development and health. The in situ DNA sequencing and sample preparation with real-time data analysis and Interpretation is highly advantageous and time-efficient. The Oxford nanopore portable system is the latest sequencing technology used for current metagenomic analysis. This development in sequencing technologies is a token of their rate of evolution. Such action is currently being used in some of the most challenging parts of the Earth, where the heavy machinery of NGS is impossible to transport; studying its metagenome would be impossible without the Oxford Nanopore technology. One study involved DNA sequencing in the Antarctic dry valley region, where environmental samples were used to perform metagenomic analyses using a completely portable sequencer and allied tools [103].

The study of the rhizosphere is only the tip of the ice burg; so many other parts of the plant can reveal unimaginable concepts and strategies we humans can use for the betterment of agriculture. The example of the pea plant rhizosphere gives the amplitude of information that can be predicted using a metagenomic approach. Here we attempted to simplify the metagenomic elements and their catalytic role in understanding the relationship between the rhizosphere and the crops. Analysis and Interpretation of the metagenomic data is a whole new world because of the heavy quantity and quality of bioinformatics used in them. The fact is that these tools and software are program-driven; there will always be new creativity in these tools to improve the understanding of data and minimize the manual inputs required in the process.

Furthermore, this topic does not end here but is only the start. In our case, the rhizosphere can be further studied using an integrated multi-OMICS approach called the new branch of System Biology [104]. It is an interdisciplinary field involving complex interactions in the biological world and can extract information starting from the genome (metagenomic), mRNA (metatranscriptomics), protein (metaproteome), and metabolites (metametabolome) [105].

## Data Availability

All the data and literature associated with this study were taken from publicly available resources. All are cited in the manuscript text.

## Abbreviations

NGS: Next Generation Sequencing
OUT: Operational taxonomic units
ITS: Internal transcribed spacer
QC: Quality Control
qPCR: Quantitative Polymerase Chain Reaction
