# Correction: Analysis and Interpretation of Metagenomics Data: An Approach

**DOI:** 10.1186/s12575-024-00235-4

**Published:** 2024-04-04

**Authors:** Gauri S. Navgire, Neha Goel, Gifty Sawhney, Mohit Sharma, Prashant Kaushik, Yugal Kishore Mohanta, Tapan Kumar Mohanta, Ahmed Al-Harrasi

**Affiliations:** 1 Department of Microbiology, Savitribai Phule Pune University, Pune, Maharashtra 411007 India; 2 Department of Genetics and Tree Improvement, Forest Research Institute, Dehradun, 248006 India; 3 Inflammation Pharmacology Division, Academy of Scientific and Innovative Research (AcSIR), CSIR-Indian Institute of Integrative Medicine, Jammu-180001, Jammu Kashmir India; 4 Department of Molecular Medicine, Medical University of Warsaw and Malopolska Center of Biotechnology, Krakow, Poland; 5 Independent Researcher, Valencia, Spain; 6 University of Science and Technology Meghalaya, Baridua, Meghalaya 793101 India; 7 Natural and Medical Sciences Research Center, University of Nizwa, Nizwa, 616 Oman

**Correction: Biol Proced Online 24:18, (2022)**

10.1186/s12575-022-00179-7

Following the publication of the original article [1], the authors regret to inform that there was an error in the affiliation details for Prashant Kaushik. The authors apologize for any inconvenience caused by this error. Prashant Kaushik should have been listed as “Independent Researcher”. The author details have been updated in the online version of the paper. The authors affirm that this correction does not affect the scientific conclusions of the research. The original publication has been updated accordingly.
